# Design and Processing of Novel PBS/PVOH Blown Films for Food Packaging: Effect of PVOH Phase Structuring on Morphology and Functional Performance

**DOI:** 10.3390/polym18111367

**Published:** 2026-05-31

**Authors:** Antonio Barbato, Francesco Palmieri, Emilia Garofalo, Annalisa Apicella, Loredana Incarnato, Luciano Di Maio

**Affiliations:** 1 Department of Industrial Engineering, University of Salerno, Via Giovanni Paolo II, 132, 84084 Fisciano, SA, Italy; abarbato@unisa.it (A.B.); egarofal@unisa.it (E.G.); anapicella@unisa.it (A.A.); lincarnato@unisa.it (L.I.); ldimaio@unisa.it (L.D.M.); 2 National Interuniversity Consortium of Materials Science and Technology (INSTM), Via G. Giusti, 9, 50121 Florence, FI, Italy

**Keywords:** poly(butylene succinate), poly(vinyl alcohol), biodegradable blends, blown films, food packaging, barrier properties, phase morphology, rheology, gel fraction, structure–property relationships

## Abstract

Biodegradable polymer blends are promising materials for flexible packaging films with tunable properties. In this work, poly(butylene succinate)/poly(vinyl alcohol) (PBS/PVOH) blown films were produced by twin-screw melt compounding followed by film blowing, and the effect of PVOH content on phase organization, processability, morphology, and functional performance was investigated. The blends showed phase-separated morphologies and composition-dependent structural evolution. DSC indicated that both polymers largely retained their crystallization ability, although the crystallinity decrease was more evident for PVOH. Rheological analysis revealed limited compatibility and increasing elastic response at higher PVOH contents, consistent with the formation of structured PVOH insoluble gel-like domains. SEM confirmed droplet–matrix morphologies, becoming coarser and more heterogeneous at high PVOH content, with film-blowing instability for PBS/PVOH 20/80. PVOH incorporation improved oxygen and water-vapor barrier properties and increased stiffness, but progressively reduced ductility. Model fitting supported the structure–property correlations, relating film performance to blend composition, morphology, and PVOH phase organization. Among the processable formulations, PBS/PVOH 80/20 showed the best balance between improved barrier properties and acceptable extensibility for food packaging application. Overall, PBS/PVOH blown films are promising biodegradable systems for flexible food packaging, provided that PVOH phase structuring is properly controlled.

## 1. Introduction

Recent European policy initiatives, including the Packaging and Packaging Waste Regulation (PPWR, Regulation (EU) 2025/40), are accelerating the transition toward more sustainable packaging solutions. In this framework, biodegradable polymers such as poly(lactic acid) (PLA), poly(butylene succinate) (PBS), poly(butylene adipate-co-terephthalate) (PBAT), and polyhydroxyalkanoates (PHAs) are attracting increasing attention for food-packaging applications as alternatives to conventional fossil-based plastics [1,2,3,4]. However, their replacement requires a suitable balance of processability, mechanical performance, thermal stability, and barrier properties, which is rarely achieved by a single biodegradable polymer. Therefore, several strategies, including plasticization, copolymerization, reactive modification, filler or nanoparticle incorporation, and polymer blending, have been explored to tailor the performance of biodegradable materials [5,6,7,8,9,10,11]. Among them, melt blending is particularly attractive because it combines complementary polymer properties using conventional, scalable, and cost-effective technologies, such as extrusion and film blowing, while avoiding complex synthetic routes and solvent-based procedures [12,13]. Accordingly, biodegradable blends based on PLA, PBS, PBAT, and PHAs have been widely investigated to improve mechanical, thermal, impact, and barrier performance [8,14,15,16]. However, their final properties depend strongly on composition, processing conditions, miscibility, phase morphology, interfacial adhesion, and crystallization behavior [17,18,19]. Thus, high-performance biodegradable blends require both suitable polymer combinations and a clear understanding of processing-induced structural changes and their impact on functional response.

Among biodegradable polymers, PBS and PVOH are particularly interesting because of their complementary characteristics. PBS is a commercially relevant biodegradable polyester with good melt processability, balanced mechanical strength and flexibility, and suitability for extrusion and blown-film production [20,21]. However, neat PBS generally shows insufficient oxygen-barrier performance, and this limitation can restrict its use in food-packaging applications where protection against oxidation is required [22]. Conversely, PVOH is known for its high polarity and excellent oxygen-barrier performance, especially under dry conditions, although its melt processing remains challenging because of brittleness, moisture sensitivity, and a narrow thermal-processing window [23,24,25,26]. Moreover, owing to its high crystallinity and strong inter- and intramolecular hydrogen bonding, PVOH can form ordered domains when chain mobility and local PVOH concentration are sufficiently high [27,28,29]. Therefore, in PBS/PVOH blends, the PVOH phase may therefore play a dual role: improving barrier performance while also promoting melt structuring and morphology instability when its local organization becomes excessive. Thus, PBS/PVOH blends are attractive but challenging systems for flexible packaging, as they aim to combine the processability and toughness of PBS with the excellent oxygen-barrier performance of PVOH.

From a miscibility standpoint, PBS/PVOH blends belong to the broader class of systems combining biodegradable polyesters with highly polar hydroxyl-rich polymers. Owing to differences in polarity and intermolecular interactions, these systems generally show limited miscibility, often resulting in phase separation, weak interfacial adhesion, and reduced ductility [18,19].

Similar behavior has been reported for other PVOH/biodegradable polyester blends. In PLA/PVOH systems, the polarity and hydrogen-bonding ability of PVOH strongly affect processing and mechanical response, leading to composition-dependent structures ranging from dispersed domains to island-like or co-continuous morphologies. Several plasticization strategies involving lacti-glyceride, monobutyl maleate, glycerol, and lactic acid, have been investigated to improve processability and interfacial response [30,31,32,33,34]. In addition, reactive compatibilizers, tailored copolymers, and interfacial modifiers have been used to reduce interfacial tension, refine the dispersed phase, and improve stress transfer in immiscible PVOH/biodegradable polyester blends [18,19,35,36]. For example, methylene diphenyl diisocyanate (MDI) and polymeric methylene diphenyl diisocyanate (pMDI) have been used in PBAT/EVOH blends to improve interfacial adhesion and morphology refinement [37,38].

By contrast, direct studies on PBS/PVOH systems remain very limited, especially for flexible films produced by melt compounding and film blowing [21,39,40]. To the best of our knowledge, the available PBS/PVOH-related literature mainly concerns non-packaging systems, such as PVOH/PBS conductive and reinforced hydrogels [41,42] and agricultural composite materials [43]. Accordingly, the miscibility, morphology development, processability limits, and functional performance of PBS/PVOH blown films remain insufficiently clarified.

In this context, our preliminary study demonstrated the feasibility of producing PBS-rich PBS/PVOH biodegradable films by melt blending and blown-film extrusion, highlighting the beneficial effect of PVOH on the barrier performance of PBS-based systems [40]. Building on these findings, the present work extends the investigated composition range toward PVOH-rich blends, namely 100/0, 80/20, 60/40, 40/60, 20/80, and 0/100 wt%, to clarify how the composition affects morphology development, processability, and functional performance. Pelletized materials were first characterized by differential scanning calorimetry (DSC), dynamic rheology, and insoluble PVOH fraction measurements to assess thermal behavior, crystallinity, blend compatibility, melt elasticity, and the possible formation of structured PVOH domains. Subsequently, blown films were produced and analyzed by FTIR spectroscopy, SEM, oxygen and water-vapor permeability, tensile testing, and UV–Vis spectroscopy to relate phase interactions and morphology to final properties. Literature models were also applied to permeability and mechanical data as interpretative tools supporting the proposed structure–property correlations. Overall, the novelty of this work lies in the systematic investigation of a scarcely explored PBS/PVOH blown-film system over a broad composition range, and in correlating PVOH phase organization with processability, morphology, barrier properties, optical response, and mechanical performance.

## 2. Materials and Methods

### 2.1. Materials

Poly(butylene succinate) (PBS, grade FZ91; density 1.26 g/cm^3^; melting temperature 115 °C) was supplied by Mitsubishi Chemical Corporation (Tokyo, Japan). Poly(vinyl alcohol) (PVOH, grade Mowiflex LP002; bulk density 0.6–0.9 g/cm^3^; melting temperature 210–220 °C), an experimental extrusion-grade PVOH specifically developed for thermoplastic processing, was provided by Kuraray Europe GmbH (Hattersheim am Main, Germany). Chloroform (CAS No. 67-66-3) and distilled water (CAS No. 7732-18-5) were purchased from Sigma-Aldrich (St. Louis, MO, USA). All the organic solvents utilized were analytical grade.

### 2.2. Blending and Blown Films Production

PBS and PVOH pellets were dried under vacuum at 80 °C for 14 h. The dried materials were then melt compounded at different PBS/PVOH weight ratios (100/0, 80/20, 60/40, 40/60, 20/80, and 0/100 wt%) using a Collin ZK 25-48D co-rotating twin-screw extruder (25 mm screw diameter, L/D = 42), operated at a screw speed of 200 rpm and a constant temperature profile of 220 °C. This processing temperature was selected as it is sufficiently above the melting temperatures of both polymers, while remaining below their degradation onset temperatures, which are approximately 349 °C for PBS and 301 °C for PVOH, as reported in the Supplementary Materials, Table S1. The extrudate, obtained through a round die, was air-cooled and pelletized. Before film blowing, neat PBS, neat PVOH, and all blended pellets were dried again under vacuum. Film samples were subsequently produced on a GIMAC single-screw extruder (12 mm screw diameter, L/D = 24), operated with a flat temperature profile of 220 °C from hopper to die. The blown film die, equipped with a radial spiral mandrel distributor, had an inner diameter of 30 mm ($D_{D})$ and a die gap (S_G_) of 0.8 mm. A Teach-Line take-up unit (Collin) was employed to inflate the bubble and draw films. Table 1 summarizes the sample coding, the average film thickness (l), and the corresponding BUR, TUR, and TUR/BUR values. The blow-up ratio (BUR), which describes the extent of transverse stretching of the bubble, was defined as

(1) $$BUR=\frac{D_{B}}{D_{D}}$$

where $D_{B}$ represent the bubble diameter and $D_{D}$ the die inner diameter (30 mm). Here, $D_{B}$ was calculated from the lay-flat width (*LFW*) according to Equation (2):

(2) $$D_{B}=\frac{2\;\cdot LFW\;}{\pi }$$

TUR, which represents the extent of longitudinal drawdown during film formation, was calculated according to Equation (3):

(3) $$TUR=\frac{S_{G}\;}{l\cdot BUR}$$

As can be observed from Table 1, comparable TUR/BUR values (ranging from 5.3 to 7) were achieved among the collected samples indicating that the films were produced under similar stretching conditions, thus allowing a more reliable comparison of their structure and properties.

No processing parameters are reported for the PBS/PVOH 20/80 composition, as the PBS/PVOH 20/80 composition did not yield a continuous blown film under the selected processing conditions. Only a few small fragments were collected, allowing FTIR and SEM analyses, whereas no functional performance characterization could be performed because of the high fragility of the sample.

### 2.3. Thermal Analysis by DSC

Thermal analysis of the pellets was performed using a Mettler-Toledo differential scanning calorimeter model 822 (Mettler Toledo, Columbus, OH, USA). The thermal program consisted of an heating scan from 30 to 240 °C at 10 °C/min, followed by an isothermal step at 240 °C for 5 min. The degree of crystallinity of the PBS and PVOH phases was calculated using melting enthalpy values of 110.3 J/g (${∆H}_{m}^{0,\;PBS}$) [44] and 138.6 J/g (${∆H}_{m}^{0,\;PVOH}$) [45] for 100% crystalline PBS and PVOH, respectively, while also accounting for the weight percentage of insoluble PVOH gels ($\omega_{gel,\;PVOH}$) formed during melt blending. The crystallinity values were determined according to Equations (4) and (5), respectively.

(4) $$X_{C}^{PBS}\;[\%]=\frac{{∆H}_{m}^{PBS}-{∆H}_{cc}^{PBS}}{{∆H}_{m}^{0,PBS}\cdot \omega_{PBS}}$$

(5) $$X_{C}^{PVOH}\;[\%]=\frac{{∆H}_{m}^{PVOH}\;}{{∆H}_{m}^{0,PVOH}\cdot \omega_{PVOH}\cdot (1-\omega_{gel,\;PVOH})}$$

where ${∆H}_{m}^{PBS}$ and ${∆H}_{m}^{PVOH}$ are the melting enthalpies of the PBS and PVOH phases, respectively; $\omega_{PBS}$ e $\omega_{PVOH\;}$ are their corresponding weight fractions; and ${∆H}_{cc}^{PBS}$ is the cold crystallization enthalpy of PBS.

### 2.4. Rheological Characterization

Dynamic shear rheological measurements were performed on pellets of the neat polymers and their blends using an ARES rotational rheometer (Rheometric Scientific, New Castle, DE, USA) equipped with 25 mm diameter parallel plates. Prior to testing, all pellets were vacuum-dried at 80 °C for 14 h. Frequency sweep experiments were carried out under a nitrogen atmosphere over an angular frequency range of 0.1–100 rad/s at 220 °C. A strain amplitude of 5% was applied in all tests in order to ensure operation within the linear viscoelastic regime.

### 2.5. PVOH Insoluble Fraction Extraction and Quantification

PVOH gels were separated in two stages from PBS/PVOH and processed PVOH pellets through a two-step extraction procedure. First, 10 g of pellets were immersed in 100 mL of chloroform and stirred for 24 h in order to dissolve the PBS phase selectively. The undissolved residue was then separated by filtration using filter paper and a Büchner funnel connected to a vacuum flask. In a second step, the recovered residue was treated with distilled water at 95 °C for 2 h to dissolve the soluble PVOH fraction. The remaining insoluble fraction was subsequently isolated by centrifugation, dried in a fume hood for 2 days to remove residual bound water, and finally weighed. The fraction of recovered insoluble PVOH gel was calculated according to Equation (6):

(6) $$f_{gel,PVOH}\;[\%]=\frac{m_{PVOH,r}}{m_{PVOH,O}}\times 100$$

where $m_{PVOH,r}$ is the mass of the recovered insoluble PVOH gel and $m_{PVOH,O}$ is the initial mass of PVOH in the blend. Each extraction was performed three times for each composition, and the gel fraction values are reported as mean ± standard deviation.

### 2.6. ATR-FTIR Analyses

ATR-FTIR measurements were performed on the film samples using a Nicolet 600 FTIR spectrophotometer (Thermo Scientific, Waltham, MA, USA) equipped with a Smart Performer accessory. Spectra were recorded over the 4000–700 cm^−1^ range with a resolution of 2 cm^−1^, averaging 64 scans for each sample. Spectral normalization and peak integration were carried out using Omnic software version 9.2 (Thermo Fisher Scientific, Madison, WI, USA).

### 2.7. Scanning Electron Microscopy (SEM) and Morphological Analysis

The cross-sectional morphology of the films was investigated by scanning electron microscopy (SEM). Film specimens were cryogenically fractured in liquid nitrogen in a direction perpendicular to the film extrusion direction and then sputter-coated with a thin gold layer using an Agar Auto Sputter Coater 108A (Stansted, UK) operated at 30 mA for 160 s. The coated samples were observed with a LEO 1525 scanning electron microscope (Carl Zeiss SMT AG, Oberkochen, Germany).

The morphology of PBS/PVOH films was further evaluated by quantitative image analysis of the dispersed domains visible in the SEM micrographs using ImageJ analysis software. Domain diameters ($d_{i}$) were measured from the images and converted into radii ($r_{i}=$ $d_{i}$/2). The number-average radius ($r_{n})$ and the volume-average radius ($r_{v}$) were calculated according to Equations (7) and (8), respectively:

(7) $$r_{n}\;[\mu m]=\frac{\sum_{i}r_{i}n_{i}}{\sum_{i}n_{i}}$$

(8) $$r_{v}\;[\mu m]=\frac{\sum_{i}r_{i}^{4}n_{i}}{\sum_{i}r_{i}^{3}n_{i}}$$

where $n_{i}$ represent the number of particles with radius $r_{i}$ in μm.

The number-average radius reflects the arithmetic mean of the domain size distribution and is mainly influenced by small and medium-sized particles, whereas the volume-average radius gives greater weight to larger domains and is therefore more sensitive to coalescence phenomena. The dispersity index (Đ) was calculated as:

(9) $$Đ=\frac{r_{v}}{r_{n}}$$

This parameter was used to quantify the width of the size distribution, where values significantly higher than unity indicate increasing polydispersity.

### 2.8. Oxygen and Water Vapor Permeability

Oxygen permeability was evaluated by measuring the oxygen transmission rate (OTR) with a GTT gas permeabilimeter (Brugger Feinmechanik GmbH, Munich, Germany) according to ISO 15105-1. Measurements were carried out in triplicate at 23 °C and 0% relative humidity, under an oxygen pressure difference of 1 bar and an oxygen flow rate of 80 mL/min, using film specimens with an area of 16 cm^2^. The oxygen permeability coefficient (PO_2_) was calculated by multiplying the measured OTR (expressed in cm^3^ m^−2^ day^−1^ bar^−1^) by the film thickness (mm).

Water vapor permeability was determined using a Water Vapor Permeation Analyzer (Model 7002, Systech Illinois, Princeton, NJ, USA), equipped with a P_2_O_5_-based sensor, according to ASTM F1249-90. Tests were performed at 23 °C and 50% relative humidity on film specimens with an area of 5 cm^2^ in triplicate. The water vapor permeability coefficient (*P_WV_*) was calculated as follows:

(10) $$P_{WV}=\frac{WVTR\cdot l}{∆P}$$

where WVTR is the water vapor transmission rate (g m^−2^ day^−1^), l is the average film thickness (mm), and ΔP is the partial water vapor pressure difference (bar) across the film.

The experimental $P_{O_{2}}$ and $P_{WV}$ data were fitted with mathematical models available in the literature to describe the effect of film composition and morphology on permeability behavior. Microsoft Excel software was used for the fitting procedure. The goodness of fit was evaluated through the coefficient of determination ($R^{2}$).

### 2.9. Tensile Properties

Tensile testing of the blown films was conducted using a SANS dynamometer (Sans Testing Machine Co. Ltd., Shenzhen, China) equipped with a 100 N load cell. Rectangular specimens, 12.7 mm in width and 30 mm in gauge length, were tested according to ASTM D882. Tensile tests were performed at a crosshead speed of 3 mm/min for the determination of the elastic modulus (E), while a crosshead speed of 15 mm/min was used to determine the elongation at break ($\varepsilon_{b}$). Mechanical properties were assessed in the machine direction (MD) on at least ten specimens. The results are reported as mean ± standard deviation.

The experimental elastic modulus data were fitted using Veenstra’s model [46], which has been applied in the literature to describe the stiffness behavior of immiscible polymer blends containing a stiffer dispersed phase within a softer matrix. Microsoft Excel software was used for the fitting procedure. The goodness of fit was evaluated through *R*^2^.

### 2.10. UV–Vis Spectroscopy

UV–Vis characterization of the films was performed with a UV–Vis spectrophotometer (Agilent Cary 60, Santa Clara, CA, USA) following ASTM D1746. Rectangular specimens were mounted in the sample holder, and the optical transmittance was recorded between 200 and 800 nm. Transmittance values (T%) at 280 and 560 nm were subsequently extracted from the spectra. Each film was tested in triplicate.

## 3. Results and Discussion

### 3.1. Pellet Thermal Transitions and Crystallinity

DSC analysis was performed on the pelletized materials before film blowing to assess how melt compounding affected the thermal transitions and crystallization behavior of the PBS and PVOH phases.

**Figure 1:**
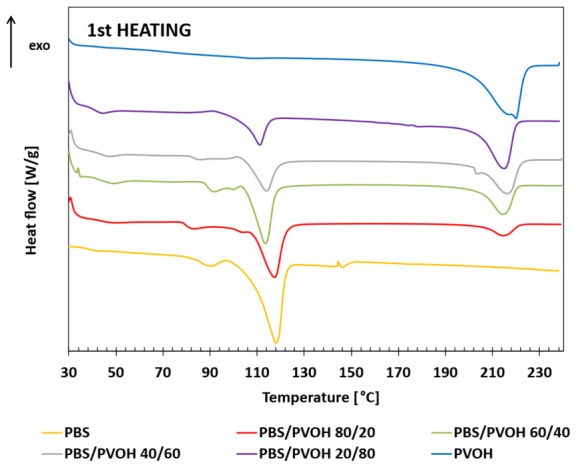
DSC thermograms obtained during the first heating scan of neat PBS, neat PVOH, and PBS/PVOH blend pellets at different compositions.

The first heating thermograms and the main DSC parameters obtained from the heating scan are reported in Figure 1 and Table 2, respectively.

Neat PBS showed the typical double-melting behavior, with endothermic peaks at 82.9 and 118.1 °C, commonly attributed to melting–recrystallization–remelting phenomena occurring during heating [47,48]. This behavior was preserved in all PBS-containing blends, indicating that the presence of PVOH did not suppress PBS crystallization. However, the main PBS melting temperature slightly decreased with increasing PVOH content, from 118.1 °C for neat PBS to 111.0 °C for the PBS/PVOH 20/80 blend, suggesting a modest reduction in crystal perfection. In addition, PBS crystallinity showed only a slight decrease with increasing PVOH content, from 67% to 65%, confirming that PBS largely retained its high crystallization ability in the blends [21]. A detectable cold-crystallization peak at 82.7 °C was observed only for PBS/PVOH 20/80, likely due to partial confinement of the minor PBS phase within the blend. This confinement may have hindered complete crystallization during cooling, allowing residual PBS chains to reorganize upon heating.

The PVOH phase exhibited a single melting transition in the high-temperature region. Neat PVOH melted at 219.8 °C, whereas the PVOH phase in the blends showed slightly lower melting temperatures, ranging from 214.2 to 216.2 °C. This decrease indicates that PVOH crystallites formed in the blends were somewhat less perfect or less thermally stable than those developed in neat PVOH. PVOH also retained a relatively high degree of crystallinity in all blend compositions, with values close to 51–55%, although lower than that of neat PVOH, which was 61%. Overall, the reduction in crystallinity was more pronounced for the PVOH phase than for the PBS phase, suggesting that PVOH crystallization was more affected by blending.

### 3.2. Dynamic Rheological Analysis

Dynamic rheological measurements were performed on the pelletized materials to evaluate the effect of blend composition on melt processability, viscoelastic response, and phase compatibility. This analysis extends our previous study on PBS/PVOH systems, where only PBS-rich compositions containing 80 and 60 wt% PBS were investigated [40]. In the present work, the rheological characterization was expanded to include the PBS/PVOH 40/60 blend, providing a clearer view of the transition from PBS-rich to PVOH-rich systems. The complex viscosity curves and Han plots are reported in Figure S1 of the Supplementary Materials. In particular, Figure S1a shows the complex viscosity as a function of angular frequency, while Figure S1b reports the corresponding Han plot, expressed as log G′ versus log G″. Reliable rheological measurements were not performed on PBS/PVOH 20/80 because incomplete stress relaxation during testing prevented stable data acquisition, suggesting severe melt structuring at very high PVOH content.

In Barbato et al. [40], the PBS/PVOH 80/20 and 60/40 blends showed lower complex viscosity than neat PBS and PVOH while retaining a PBS-like shear-thinning behavior. This reduction was attributed to poor compatibility between PBS and PVOH and to possible interfacial slippage between the two phases. Consistently, the Han plot indicated phase separation and moreover both compositions still displayed a mainly viscous, liquid-like response. This behavior suggests that, in PBS-rich compositions, the PBS matrix still mainly governs the overall melt relaxation.

The inclusion of the PBS/PVOH 40/60 composition in the present work revealed a clear change in the melt behavior. Unlike the PBS-rich blends, PBS/PVOH 40/60 showed a marked increase in complex viscosity, particularly at low angular frequencies, together with higher G′ values at comparable G″ in the Han plot. This different response does not indicate better compatibility between PBS and PVOH. Rather, it suggests that, when PVOH becomes the major component, the relaxation of the heterogeneous melt is increasingly affected by the PVOH-rich phase. The higher PVOH content may therefore promote slower relaxation phenomena and a more elastic response, possibly related to the onset of PVOH-rich phase structuring.

**Figure 2:**
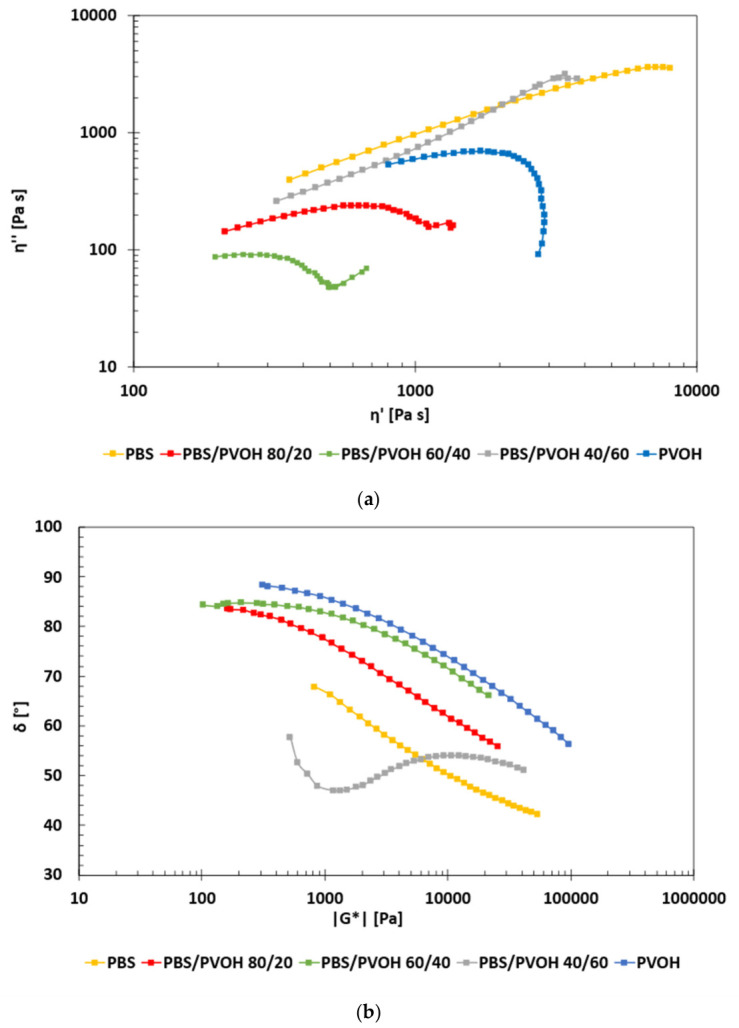
(**a**) Cole–Cole plot (η″ vs. η′) for neat PBS, neat PVOH, and PBS/PVOH blends, highlighting changes in relaxation behavior and deviations associated with blend composition. (**b**) Van Gurp–Palmen plot (phase angle δ as a function of complex modulus |G*|) for the same systems, showing the evolution of viscoelastic response and indicating modifications in phase structure and intermolecular interactions with increasing PVOH content.

To better clarify the origin of this composition-dependent response, Cole–Cole and Van Gurp–Palmen plots were employed. Figure 2a shows the Cole–Cole plot, reported as η″ versus η′, whereas Figure 2b shows the Van Gurp–Palmen plot, reported as δ versus |G*|.

The Cole–Cole plot, reported in Figure 2a, was used to analyze the relaxation behavior of neat polymers and PBS/PVOH blends. Neat PBS and neat PVOH showed monotonic arc-like profiles, generally associated with a single dominant relaxation mechanism in homogeneous polymer melts [49,50,51]. In contrast, the PBS/PVOH blends exhibited a more complex rheological response. In PBS-rich blends, a double-arc profile was observed, which is commonly associated with phase-separated droplet–matrix systems. The contribution at lower η′ values can be related to the relaxation of the polymer phases, while the contribution at higher η′ values is associated with the relaxation of the interface involving the dispersed droplets [52,53,54]. The PBS/PVOH 40/60 blend displayed an upward trend with a clear tail at high η′ values, suggesting slow interfacial dynamics and the development of a more structured phase within the heterogeneous melt [54,55,56].

The Van Gurp–Palmen plot, reported in Figure 2b, was used as an additional tool to obtain further insight into molecular structuration and morphology development in neat polymers and PBS/PVOH blends. In this representation, linear polymer melts generally tend toward high phase angles at low complex modulus, whereas deviations from this behavior, such as a reduction in δ or the appearance of local valleys, may indicate an increased elastic contribution associated with branching, long relaxation modes, or network-like structures [49,57,58,59]. In the present case, neat PVOH showed a more linear-like response, while neat PBS was located at lower phase angles, in agreement with its higher elastic character [40,59]. The blend response reflected the development of composition-dependent structuring. In particular, PBS/PVOH 60/40 displayed a slight valley at low |G*|, suggesting the onset of slowly relaxing domains, while this feature became more pronounced for PBS/PVOH 40/60. The deeper valley observed for this composition indicates a more constrained melt structure, likely related to the organization of the PVOH phase, considering the known sensitivity of PVOH to irreversible chemical structuring phenomena. In this regard, selective extraction analyses were performed on the investigated blends to highlight the presence of insoluble fractions.

### 3.3. Insoluble Fraction Quantification

The insoluble fraction was quantified to assess whether the melt structuring suggested by rheological analysis was associated with insoluble PVOH domains. The extraction procedure was performed on processed neat PVOH and on all PBS/PVOH blend compositions, while neat PBS was reported as “not applicable” because it does not contain PVOH. The gel fraction values measured are reported in Table 3.

Gel formation was strongly composition-dependent. Neat PVOH showed only a limited insoluble fraction, equal to 2.2 ± 1.1 wt%, whereas PBS/PVOH blends exhibited progressively higher gel contents with increasing PVOH concentration, reaching 20.8 ± 3.1 wt% for PBS/PVOH 20/80. The insoluble fraction recovered from the PBS/PVOH blends was found to consist essentially of PVOH, as confirmed by the FTIR spectrum reported in Figure S2, representative of the PBS/PVOH 40/60 recovered sample.

These results suggest that the presence of the PBS phase promotes the formation of gel-like structures involving PVOH. Morphologically, this insoluble fraction can be regarded as PVOH-rich gel-like domains or aggregates embedded within the phase-separated PBS/PVOH morphology. As reported in the literature [57,59], the vinyl groups present along the chain of the commercial PBS used in this study may generate radicals at processing temperatures above 190 °C. These radicals can potentially abstract hydrogen atoms from PVOH chains, thereby activating crosslinking reactions within the PVOH phase. However, the extraction procedure only quantifies the insoluble material and does not identify the nature of the junctions responsible for its formation. Therefore, the observed gel fraction is discussed here as a PVOH-rich “gel-like” fraction, which may arise from strong physical association and/or irreversible structuring during melt processing. No direct evidence of ester-exchange reactions between PBS and PVOH was obtained from the present analysis. Further spectroscopic studies, including solid-state NMR, will be required to clarify this point. The formation of this structured fraction, particularly in PBS/PVOH 40/60 and PBS/PVOH 20/80 blends, is consistent with the reduced PVOH crystallinity observed by DSC and with the peculiar rheological behavior reported in Figure 2a,b.

### 3.4. Films Structural Analysis and Morphology

Due to the high gel content observed in the PBS/PVOH 20/80 sample, it was not possible to obtain a continuous and homogeneous blown film. For this composition, only film fragments were collected, which were therefore used exclusively for structural and morphological analyses as reported below.

FTIR analysis was performed on the blown films to investigate composition-dependent structural changes in the PBS/PVOH systems. Building on the spectral features previously reported by Barbato et al. [40] for PBS-rich blends, the present analysis was extended to the whole composition range.

**Figure 3:**
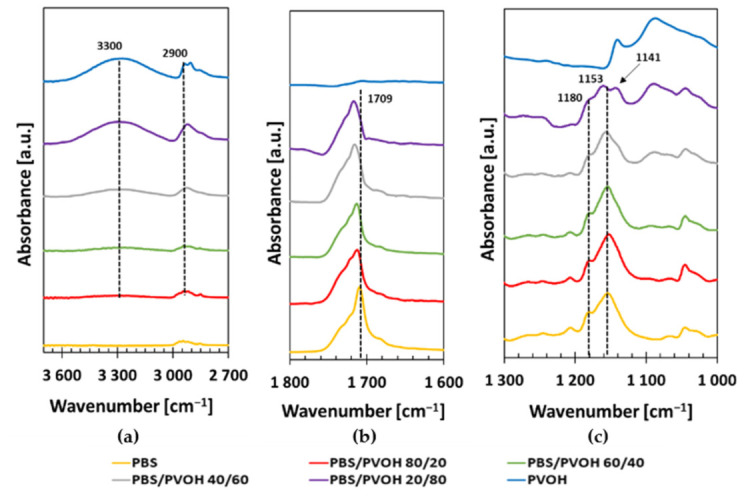
ATR-FTIR spectra of neat PBS, neat PVOH, and PBS/PVOH blend films in selected wavenumber regions: (**a**) 3700–2700 cm^−1^, highlighting the O–H stretching band of PVOH and C–H stretching bands; (**b**) 1800–1600 cm^−1^, showing the carbonyl stretching band of PBS; and (**c**) 1300–1000 cm^−1^, showing the ester-related C–O–C stretching bands of PBS.

Figure 3 reports selected magnifications of the FTIR spectra of neat PBS, neat PVOH, and PBS/PVOH blend films. In particular, Figure 3a shows the 3700–2700 cm^−1^ region, where the broad O–H stretching band of PVOH around 3300 cm^−1^ and the C–H stretching bands around 2900 cm^−1^ are visible. Figure 3b focuses on the 1800–1600 cm^−1^ region, dominated by the carbonyl stretching of PBS, located at 1709 cm^−1^ in the neat PBS film. Finally, Figure 3c shows the 1300–1000 cm^−1^ region, where the ester-related C–O–C stretching bands of PBS are detected, particularly around 1180 and 1153 cm^−1^, in agreement with the characteristic FTIR absorptions reported for PBS-based films [39].

**Figure 4:**
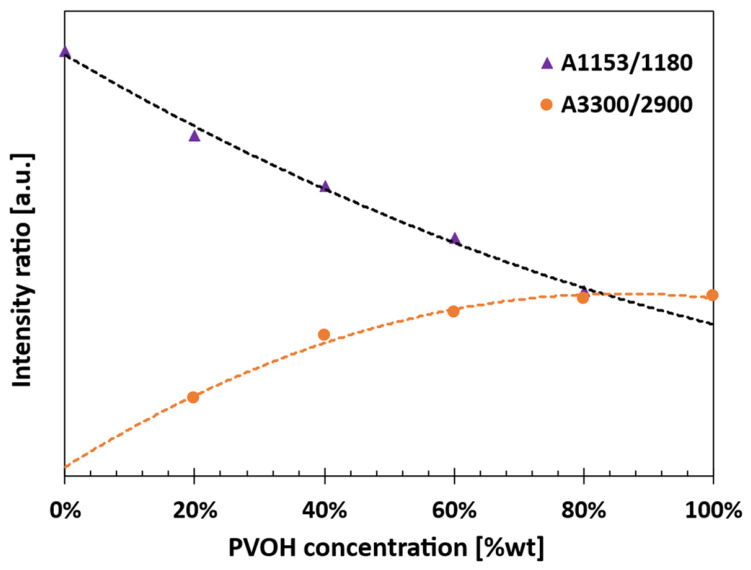
FTIR intensity ratios as a function of PVOH content in PBS/PVOH blend films: A_3300_/A_2900_, associated with the relative contribution of PVOH hydroxyl groups, and A_1153_/A_1180_, related to changes in the ester C–O–C stretching region of PBS.

To better compare the spectral changes, two intensity ratios were calculated and reported in Figure 4. The A_3300_/A_2900_ ratio was used to follow the relative contribution of PVOH hydroxyl groups, whereas the A_1153_/A_1180_ ratio was selected to monitor changes in the ester-related bands of PBS.

The A_3300_/A_2900_ ratio progressively increased with increasing PVOH content, reflecting the growing contribution of hydroxyl groups in the blends. This increase mainly follows the higher amount of PVOH, but it is also consistent with the presence of a progressively more associated PVOH phase, where inter- and intramolecular hydrogen bonding becomes increasingly relevant.

At the same time, the PBS carbonyl band showed a slight shift from 1709 cm^−1^ in neat PBS to higher wavenumbers in the blends (Figure 3b), suggesting a composition-dependent change in the local environment of PBS carbonyl groups. This variation may be related to changes in PBS chain packing and crystalline organization within the phase-separated morphology, in agreement with the slight decrease in PBS melting temperature and crystallinity observed by DSC.

Similarly, the progressive decrease in the A_1153_/A_1180_ ratio with increasing PVOH content indicates changes in the vibrational environment of PBS ester groups [60], suggesting that the PBS phase becomes increasingly affected by the presence of the more rigid and highly associated PVOH domains.

**Figure 5:**
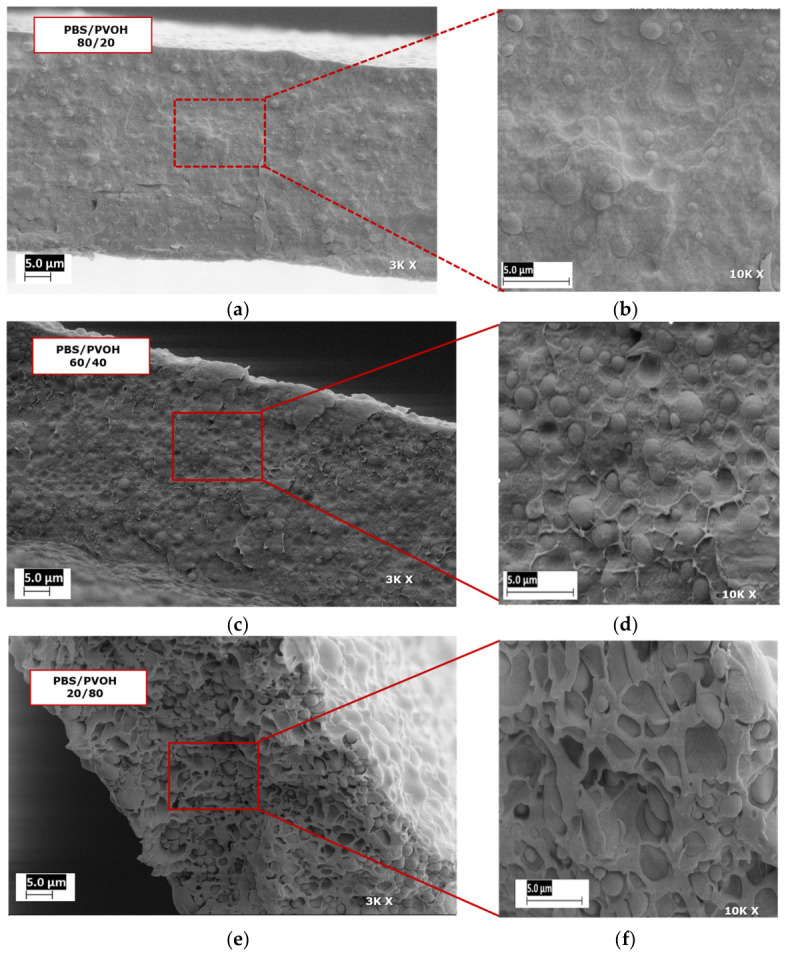
SEM micrographs of cryo-fractured cross sections of PBS/PVOH blend films at two magnifications: PBS/PVOH 80/20 at (**a**) 3k× and (**b**) 10k×; PBS/PVOH 60/40 at (**c**) 3k× and (**d**) 10k×; and PBS/PVOH 20/80 at (**e**) 3k× and (**f**) 10k×.

SEM analysis was carried out on PBS/PVOH 80/20, 60/40, and 20/80 samples, representative of low, intermediate, and high PVOH contents, respectively. Cross-sectional micrographs were used to assess phase distribution and domain size, which are expected to influence the mechanical, barrier, and optical properties discussed below. Figure 5 reports the SEM images, while Table 4 summarizes the number-average radius $\left(r_{n}\right)$, volume-average radius $\left(r_{v}\right)$, and dispersity index $\left(Đ\right)$ obtained from image analysis.

The PBS/PVOH 80/20 film showed a typical droplet–matrix morphology, with small domains dispersed in the PBS-rich matrix, in agreement with the phase-separated behavior suggested by rheological analysis A similar phase-separated morphology was observed for intermediate composition PBS/PVOH 60/40, although the higher PVOH content made the dispersed phase more evident.

In contrast, PBS/PVOH 20/80 showed a markedly coarser droplet-like morphology, with larger, irregular, and partially coalesced domains. The presence of voids and cavities, clearly visible at higher magnification, further indicates a heterogeneous and poorly compact phase morphology at high PVOH content.

Quantitative image analysis supports this trend. PBS/PVOH 80/20 and 60/40 showed similar $r_{n}$ values of 0.63 µm and low $r_{v}$ values of 0.96 and 0.85 µm, respectively, indicating relatively fine domain distributions. In contrast, PBS/PVOH 20/80 exhibited higher $r_{n}$ and $r_{v}$ values, equal to 1.63 and 4.16 µm, with a dispersity index of 2.55. This indicates a broader and more heterogeneous morphology, dominated by larger and partially coalesced domains.

### 3.5. Functional Performance of Processable Blown Films

#### 3.5.1. Oxygen and Water Vapor Permeability Performance

**Figure 6:**
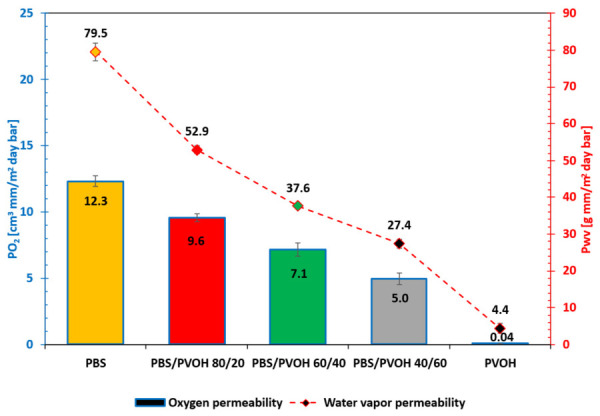
Oxygen permeability coefficient $\left(P_{O_{2}}\right)$ and water vapor permeability coefficient $\left(P_{WV}\right)$ of neat PBS, neat PVOH, and PBS/PVOH blend films as a function of composition. Values are reported as mean ± standard deviation.

Oxygen and water vapor barrier properties are key requirements for food-packaging films, since gas and moisture transfer can affect oxidation, microbial stability, texture, and shelf life. Therefore, $P_{O_{2}}$ and $P_{WV}$ were measured to evaluate the effect of PVOH incorporation on the barrier performance of PBS-based blown films. The results are reported in Figure 6, where the permeability values of neat PBS, neat PVOH, and PBS/PVOH blends are compared. No data are reported for PBS/PVOH 20/80 because the film-blowing process was unstable and only fragmented samples were collected.

In agreement with our previous work on PBS/PVOH 80/20 and 60/40 films [40], permeability progressively decreased with increasing PVOH content. Neat PBS showed a $P_{O_{2}}$ value of 12.3 cm^3^ mm m^−2^ day^−1^ bar^−1^, whereas the newly investigated PBS/PVOH 40/60 film reached 5.0 cm^3^ mm m^−2^ day^−1^ bar^−1^, corresponding to a 59% reduction. This confirms the beneficial contribution of the PVOH phase to the oxygen-barrier performance of PBS-based films due to its higher intrinsic oxygen-barrier properties. However, these measurements were performed under dry conditions; therefore, possible humidity-induced changes in oxygen permeability should be considered when evaluating these films for real food-packaging applications.

A similar trend was observed for water vapor permeability. Neat PBS showed a $P_{WV}$ value of 79.5 g mm m^−2^ day^−1^ bar^−1^, while neat PVOH showed a markedly lower value of 4.4 g mm m^−2^ day^−1^ bar^−1^. Although PVOH is generally hydrophilic and moisture-sensitive, the low $P_{WV}$ measured for the neat PVOH film can be reasonably related to the specific extrusion-grade formulation used in this work. According to information provided by the supplier, this material is based on modified PVOH grades containing ethylene units, which improve water resistance and have been shown in previous studies to maintain low oxygen/water vapor permeabilities up to about 60% RH [25,61]. The PBS/PVOH blends displayed intermediate values between those of the neat polymers, with $P_{WV}$ decreasing from 52.9 g mm m^−2^ day^−1^ bar^−1^ for PBS/PVOH 80/20 to 37.6 and 27.4 g mm m^−2^ day^−1^ bar^−1^ for PBS/PVOH 60/40 and 40/60, respectively. These values correspond to reductions of about 33%, 53%, and 65% compared with neat PBS.

Considering the $P_{O_{2}}$ and $P_{WV}$ values obtained, the developed PBS/PVOH can be regarded as improved medium-barrier biodegradable candidate films for dry or intermediate-moisture food-packaging applications, such as bakery, confectionery, and snack-type products requiring moderate oxygen and moisture control [22,62,63].

**Figure 7:**
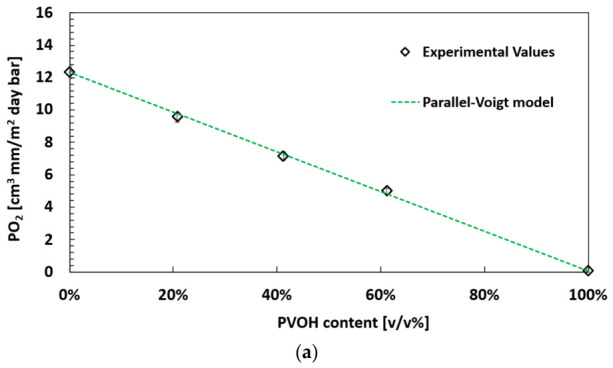

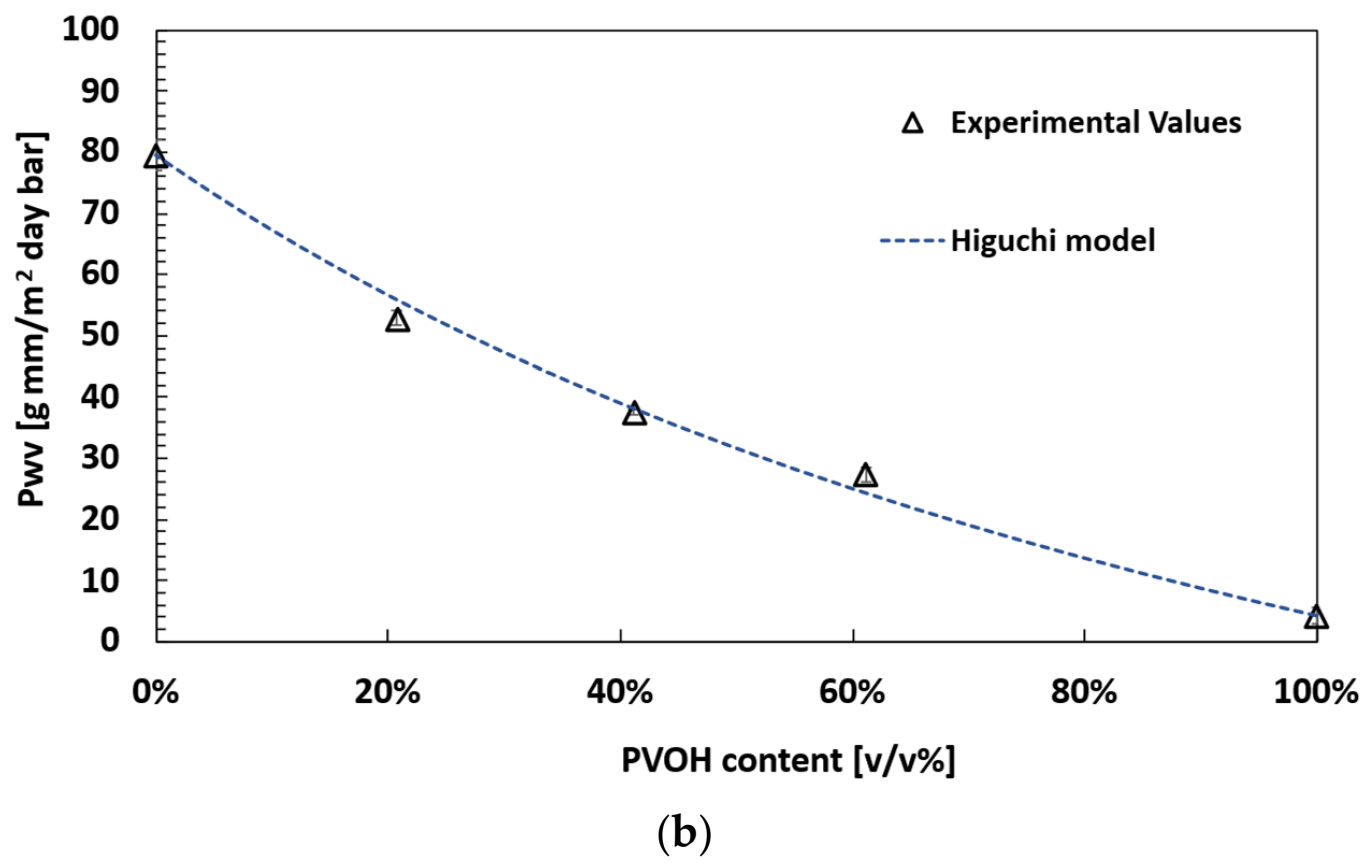
Experimental permeability values and model fitting as a function of PVOH content: (**a**) oxygen permeability coefficient $\left(P_{O_{2}}\right)$ fitted with the Parallel–Voigt model; and (**b**) water vapor permeability coefficient $\left(P_{WV}\right)$ fitted with the Higuchi model.

To better interpret the permeability behavior, the experimental $P_{O_{2}}$ and $P_{WV\;}$ values were fitted using literature models for two-phase polymer blends with droplet-like morphologies [64]. Given the limited number of blend compositions, the models were used as interpretative tools to support the structure–property analysis rather than as a definitive predictive description. The comparison between experimental data and model predictions is shown in Figure 7: $P_{O_{2}}$ was fitted with the Parallel–Voigt model, whereas $P_{WV}$ was described using the Higuchi model. The corresponding equations are reported in Equations (11) and (12), respectively:

(11) $$P=P_{c}\varphi_{c}+P_{d}\varphi_{d}$$

(12) $$P=P_{c}\left[1+\frac{3\varphi_{d}\left(\frac{P_{d}-P_{c}}{P_{d}+2P_{c}}\right)}{1-\varphi_{d}\left(\frac{P_{d}-P_{c}}{P_{d}+2P_{c}}\right)-0.78(1-\varphi_{d})\left(\frac{P_{d}-P_{c}}{P_{d}+2P_{c}}\right)}\right]$$

where *P_c_* and *P_d_* represent the permeability of the permeant in the continuous and dispersed polymer phases, respectively, while *φ_c_* and *φ_d_* denote their corresponding fractional volumes.

The Parallel–Voigt and Higuchi models provided a good fit of the experimental permeability data, while describing different transport contributions. The Parallel–Voigt model treats the overall permeability as the additive contribution of each polymer phase; therefore, the phase with higher barrier performance proportionally reduces the final permeability [64,65]. This explains why $P_{O_{2}}$ was best described by the Parallel–Voigt model $\left(R^{2}=0.999\right)$: oxygen transport is mainly governed by the intrinsic oxygen-barrier character of PVOH, and the progressive decrease in $P_{O_{2}}$ reflects its increasing fraction in the blends.

Conversely, the Higuchi model provided the best description of the $P_{WV}$ data ($R^{2}=0.997$), suggesting that water vapor transport is sensitive not only to the intrinsic permeability of the two polymers, but also to the organization of the PVOH phase. In particular, the presence of the more associated crosslinked PVOH-rich domains may contribute to the lower availability of polar sites for water interaction and contribute to slow down water vapor diffusion.

#### 3.5.2. Tensile Properties

The tensile properties of neat PBS, neat PVOH, and PBS/PVOH blend films are reported in Table 5 in terms of E and ${\;\varepsilon }_{b}$, measured in the machine direction. Blown films exhibit typically anisotropic mechanical behavior because longitudinal drawdown and bubble inflation may induce different macromolecular orientations in both machine and transverse directions [66,67]. Therefore, the absence of TD tensile data represents a limitation of this study, and the present results describe only the mechanical response along the main drawing direction. Nevertheless, since all processable films were produced under comparable stretching conditions, as indicated by the similar TUR/BUR values in Table 1, the MD data allow a consistent comparison of the effect of composition. Similar composition-dependent trends may also occur in TD, although this needs experimental confirmation in future work. Representative stress–strain curves of the films are reported in Figure S3 of the Supplementary Materials, providing further insight into the composition-dependent deformation behavior.

Neat PBS showed an elastic modulus of 466 ± 49 MPa and an elongation at break of 254 ± 30%, confirming its ductile behavior. Conversely, neat PVOH exhibited a much higher modulus, equal to 1459 ± 12 MPa, and a lower elongation at break of 35 ± 9%, in agreement with its more rigid and brittle character [68,69,70].

The addition of PVOH progressively increased the stiffness of PBS-based films. In particular, the elastic modulus increased from 466 MPa for neat PBS to 597, 689, and 879 MPa for PBS/PVOH 80/20, 60/40, and 40/60, respectively.

To further interpret the stiffness evolution, the experimental elastic modulus values along MD were compared with Veenstra’s model, which is commonly used to describe polymer blends containing a stiffer dispersed phase within a softer matrix [46] (Equation (13)). In the present case, PVOH was considered as the stiffer phase $\left(E_{1}\right)$, while PBS was considered as the softer matrix $\left(E_{2}\right)$.

(13) $$E=\left(1-\lambda^{2}\right)E_{2}+\frac{\lambda^{2}E_{1}E_{2}}{\lambda E_{2}+(1-\lambda )E_{1}}$$

where $\lambda =\sqrt[{3}]{\varphi_{d}}=\sqrt[{3}]{1-\varphi_{m}}$; $\varphi_{d}$ and $\varphi_{m}$ are the volume fractions of the dispersed phase and matrix, respectively, while $E_{1}$ and $E_{2}$ are the elastic moduli of polymers 1 and 2, respectively.

**Figure 8:**
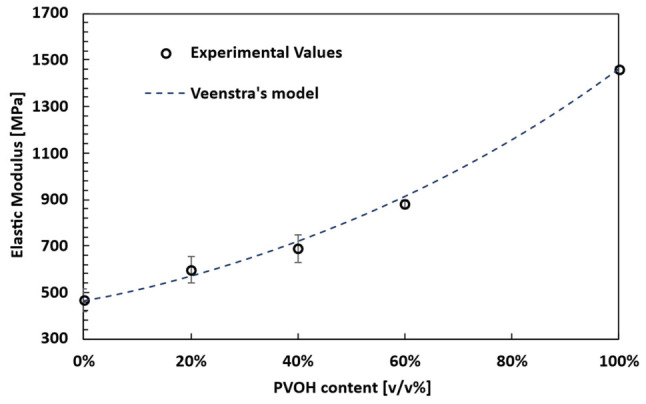
Experimental elastic modulus of neat PBS, neat PVOH, and PBS/PVOH blend films as a function of PVOH content, compared with the theoretical prediction obtained from Veenstra’s model. Values are reported as mean ± standard deviation.

The comparison between experimental data and Veenstra’s model is reported in Figure 8. The good agreement obtained $\left(R^{2}=0.995\right)$ supports the interpretation that the increase in elastic modulus is mainly governed by the growing contribution of the stiffer PVOH phase within the phase-separated PBS/PVOH morphology.

However, the increase in stiffness was accompanied by a progressive loss of ductility. The elongation at break decreased from 254% for neat PBS to 75% for PBS/PVOH 80/20, a value still broadly acceptable for flexible food-packaging films requiring deformability during handling and converting [71]. At higher PVOH contents, ductility sharply dropped to 15% and 6% for PBS/PVOH 60/40 and 40/60, respectively. Since neat PVOH showed an elongation at break of 35%, this collapse can be reasonably attributed to the phase-separated morphology, and in turn to the limited stress transfer across the interface, as well as to the presence of structured PVOH-rich domains acting as stress-concentration points.

#### 3.5.3. Optical Properties

**Figure 9:**
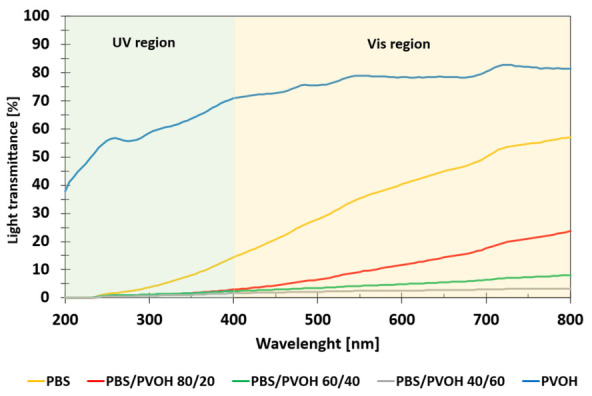
UV–Vis transmittance spectra of neat PBS, neat PVOH, and PBS/PVOH blend films in the 200–800 nm wavelength range, highlighting the UV and visible regions.

The optical properties of the films were evaluated by UV–Vis spectroscopy, since light transmission is relevant for food-packaging applications both in terms of product visibility and protection against photo-oxidative degradation. The UV–Vis spectra are reported in Figure 9, while the transmittance values at 280 nm and 560 nm are summarized in Table 6.

Neat PVOH showed the highest transparency, with a $T_{560}$ value of 79.3%, whereas neat PBS displayed a lower visible transmittance of 36.7%. The incorporation of PVOH into PBS led to a marked decrease in transparency, with $T_{560}$ values of 9.8, 4.5, and 2.5% for PBS/PVOH 80/20, 60/40, and 40/60, respectively. As PVOH content increases, dispersed domains, interfacial heterogeneities, and gel-like domains become more relevant, leading to lower visible transmittance [72,73].

Conversely, all PBS/PVOH blends showed very low transmittance at 280 nm, with $T_{280}$ values close to or below 1%. Although this behavior limits film transparency, it provides an interesting UV-shielding effect, which can be advantageous for food-packaging applications where protection against UV-induced oxidation is required. Therefore, the optical response of PBS/PVOH films reflects a trade-off between reduced visible transparency and enhanced UV protection. This behavior may be advantageous for applications where light shielding is preferred over high transparency, particularly for products sensitive to UV-induced oxidation [61].

## 4. Conclusions

This work demonstrated the feasibility of producing PBS/PVOH blown films by melt blending and film blowing, while highlighting the critical role of PVOH phase organization in controlling processability, morphology, and final properties.

DSC showed that both polymers largely retained their crystallization ability and maintained high crystallinity values, although a decrease in crystallinity was observed, more pronounced for PVOH than for PBS. Rheological analysis revealed limited PBS/PVOH compatibility and increasing elastic response at higher PVOH contents, in agreement with the growth of the insoluble PVOH fraction and the development of structured PVOH-rich domains.

FTIR showed composition-dependent structural changes without new absorption bands, indicating that PBS and PVOH preserved their chemical identity in phase-separated blends. SEM confirmed droplet–matrix morphologies. PBS/PVOH 80/20 and 60/40 showed relatively fine and comparable domain distributions, whereas PBS/PVOH 20/80 displayed a marked morphological coarsening, with larger, more polydisperse, and partially coalesced domains. The PBS/PVOH 20/80 composition was the most critical, showing film-blowing instability, high gel fraction, large partially coalesced domains, and voids.

PVOH incorporation progressively improved the barrier performance of PBS-based films, reducing both oxygen and water-vapor permeability. The oxygen permeability trend was well described by the Parallel–Voigt model, confirming the dominant role of the intrinsic barrier properties PVOH phase, while the Higuchi model indicated that water-vapor transport was also affected by blend morphology and PVOH phase organization. The increase in elastic modulus with PVOH content was well fitted by Veenstra’s model, highlighting the contribution of the rigid PVOH phase to film stiffening. However, the barrier improvement was accompanied by a progressive loss of ductility at higher PVOH contents, mainly related to phase separation, limited stress transfer, and structured PVOH-rich domains acting as stress-concentration points.

Among the processable compositions, PBS/PVOH 80/20 showed the best balance between improved barrier performance and acceptable ductility. This formulation appears most suitable for dry or low-moisture food packaging, such as bakery, confectionery, and snack-type products, where moderate oxygen and moisture control is required.

Overall, PBS/PVOH blends are promising biodegradable systems for flexible packaging, provided that PVOH phase structuring is controlled. For higher PVOH contents, compatibilization and/or plasticization will be needed to improve processability and ductility while preserving the barrier enhancement. Future work should therefore focus on morphology stabilization, reduction of irreversible gel formation, and optimization of film performance.

## Figures and Tables

**Table 1:** Sample coding, average film thickness, and corresponding processing parameters of the blown films, expressed in terms of blow-up ratio (BUR), take-up ratio (TUR), and TUR/BUR ratio.

Film Sample	Thickness [µm]	BUR	TUR	TUR/BUR
PBS	42 ± 2	1.7	11.5	7
PBS/PVOH 80/20	43 ± 1	1.9	10.0	5.3
PBS/PVOH 60/40	42 ± 2	1.7	10.9	6.3
PBS/PVOH 40/60	43 ± 2	1.8	10.2	5.6
PBS/PVOH 20/80	n.a.	n.a.	n.a.	n.a.
PVOH	40 ± 3	1.8	11.2	6.3

“n.a.” indicates not available or not detectable.

**Table 2:** Thermal properties of neat PBS, neat PVOH, and PBS/PVOH blends referred to the first heating determined by DSC. Reported values include melting temperatures (T_m1_, T_m2_ for PBS and T_m_ for PVOH), melting enthalpies (ΔH_m1_, ΔH_m2_, ΔH_m_), cold crystallization temperature (Tcc), cold crystallization enthalpy (ΔHcc), and the degree of crystallinity of the individual phases (Xc). “n.a.” indicates not available or not detectable.

Pellet Sample	PBS							PVOH		
	T_m1_ [°C]	ΔH_m1_ [J/g]	T_cc_ [°C]	ΔH_cc_ [J/g]	T_m2_ [°C]	ΔH_m2_ [J/g]	X_c_ [%]	T_m_ [°C]	ΔH_m_ [J/g]	X_c_ [%]
PBS	82.9	5.2	n.a.	n.a.	118.1	68.7	67.2	n.a.	n.a.	n.a.
PBS/PVOH 80/20	82.4	8.1	n.a.	n.a.	117.5	51.0	67.2	214.5	14.8	53.7
PBS/PVOH 60/40	91.6	6.0	n.a.	n.a.	113.6	37.6	66.0	214.2	29.9	54.9
PBS/PVOH 40/60	85.7	1.1	n.a.	n.a.	114.4	27.3	64.5	216.2	40.7	52.3
PBS/PVOH 20/80	n.a.	n.a.	82.7	2.0	111.0	16.5	65.5	214.9	47.4	51.2
PVOH	n.a.	n.a.	n.a.	n.a.	n.a.	n.a.	n.a.	219.8	82.2	60.7

**Table 3:** PVOH gel fraction, $f_{gel,PVOH}$, measured for neat PVOH and PBS/PVOH blends with different compositions after melt processing.

Pellet Sample	$f_{gel,PVOH}\;[\%]$
PBS	n.a.
PBS/PVOH 80/20	3.3 ± 1.2
PBS/PVOH 60/40	4.2 ± 2.0
PBS/PVOH 40/60	10.7 ± 1.7
PBS/PVOH 20/80	20.8 ± 3.1
PVOH	2.2 ± 1.1

“n.a.” indicates not applicable.

**Table 4:** Number-average radius $\left(r_{n}\right)$, volume-average radius $\left(r_{v}\right)$, and dispersity index $\left(Đ=r_{v}/r_{n}\right)$ of the dispersed domains measured from SEM micrographs of representative PBS/PVOH blend films.

Film Sample	$r_{n}\;[\mu m]$	$r_{v}\;[\mu m]$	Đ [−]
PBS/PVOH 80/20	0.63	0.96	1.53
PBS/PVOH 60/40	0.63	0.85	1.34
PBS/PVOH 20/80	1.63	4.16	2.55

**Table 5:** Tensile properties of neat PBS, neat PVOH, and PBS/PVOH blend films measured in the machine direction: elastic modulus $\left(E\right)$ and elongation at break $\left(\varepsilon_{b}\right)$. Values are reported as mean ± standard deviation.

Film Sample	E [MPa]	$\varepsilon_{b}\;[\%]$
PBS	466 ± 49	254 ± 30
PBS/PVOH 80/20	597 ± 56	75 ± 15
PBS/PVOH 60/40	689 ± 60	15 ± 2
PBS/PVOH 40/60	879 ± 20	6 ± 1
PVOH	1459 ± 12	35 ± 9

**Table 6:** UV–Vis transmittance values of neat PBS, neat PVOH, and PBS/PVOH blend films at 280 nm $\left(T_{280}\right)$ and 560 nm $\left(T_{560}\right)$. Values are reported as mean ± standard deviation.

Film Sample	$T_{280}\;[\%]$	$T_{560}\;[\%]$
PBS	2.6 ± 1	36.7 ± 5.8
PBS/PVOH 80/20	0.8 ± 0.04	9.8 ± 0.7
PBS/PVOH 60/40	1.1 ± 0.04	4.5 ± 0.1
PBS/PVOH 40/60	0.6 ± 0.05	2.5 ± 0.3
PVOH	61.5 ± 1	79.3 ± 5.1

## Data Availability

The data presented in this study are available upon request from the corresponding author.

## Supplementary Materials

The following supporting information can be downloaded at: https://www.mdpi.com/article/10.3390/polym18111367/s1, Table S1: Thermal degradation parameters of PBS, PVOH, and PBS/PVOH blend films, showing the onset degradation temperature (Tonset) and the maximum degradation temperature from DTG analysis (T DTG max). Figure S1: Dynamic rheological response of neat PBS, neat PVOH, and PBS/PVOH blends at 220 °C: (a) complex viscosity as a function of angular frequency and (b) Han plot expressed as log G′ versus log G″, Figure S2: FTIR spectrum of the insoluble residue recovered from the PBS/PVOH 40/60 blend. The characteristic absorption bands confirm that the insoluble fraction consists essentially of PVOH, showing the main signals attributable to –OH, C–H, and C–O groups typical of poly(vinyl alcohol), Figure S3: Representative stress–strain curves of neat PBS, neat PVOH, and processable PBS/PVOH blend films measured in the machine direction according to ASTM D882.

## Abbreviations

The following abbreviations are used in this manuscript:

PPWR	Packaging and Packaging Waste Regulation
PLA	poly(lactic acid)
PBS	poly(butylene succinate)
PBAT	poly(butylene adipate-co-terephthalate)
PHAs	polyhydroxyalkanoates
PVOH	polyvinyl alcohol
MDI	methylene diphenyl diisocyanate
pMDI	polymeric methylene diphenyl diisocyanate
DSC	Differential scanning calorimetry
ATR-FTIR	Attenuated total reflectance Fourier-transform infrared spectroscopy
FTIR	Fourier-transform infrared spectroscopy
SEM	Scanning electron microscopy
UV–Vis	Ultraviolet–visible spectroscopy
BUR	Blow-up ratio
TUR	Take-up ratio
LFW	Lay-flat width
MD	Machine direction
OTR	Oxygen transmission rate
PO_2_	Oxygen permeability coefficient
P_WV_	Water vapor permeability coefficient
Tm	Melting temperature
Tcc	Cold-crystallization temperature
ΔHm	Melting enthalpy
ΔHcc	Cold-crystallization enthalpy
Xc	Degree of crystallinity
η*	Complex viscosity
η′	Real component of complex viscosity
η″	Imaginary component of complex viscosity
ω	Angular frequency
G′	Storage modulus
G″	Loss modulus
|G*|	Complex modulus
δ	Phase angle
r_n_	Number-average radius
r_v_	Volume-average radius
Đ	Dispersity index
E	Elastic modulus
ε_b_	Elongation at break
R^2^	Coefficient of determination
n.a.	Not available/not applicable
