# Supplementary material for: Design and Processing of Novel PBS/PVOH Blown Films for Food Packaging: Effect of PVOH Phase Structuring on Morphology and Functional Performance
Source: Polymers (Basel). 2026 May 31;18(11):1367. doi: 10.3390/polym18111367 (PMC13259088; doi:10.3390/polym18111367)
Supplement: Supplementary file 1 [file polymers-18-01367-s001.zip › polymers-4319536-supplementary.pdf]

Thermogravimetric analysis (TGA) was conducted on pellets of the pure materials and their blends using a Q500 analyzer from TA Instruments (New Castle, DE, USA). The analysis employed a temperature scan rate of 10°C/min over a range of 30–700°C in a nitrogen atmosphere.

Table S1. Thermal degradation parameters of PBS, PVOH, and PBS/PVOH blend films, showing the onset degradation temperature (Tonset) and the maximum degradation temperature from DTG analysis (T DTG max).

Film sample	Tonset [°C]	T DTG max [°C]
PBS	349	402
PBS/PVOH 80/20	343	398
PBS/PVOH 60/40	330	369
PBS/PVOH 40/60	328	366
PBS/PVOH 20/80	326	364
PVOH	301	372

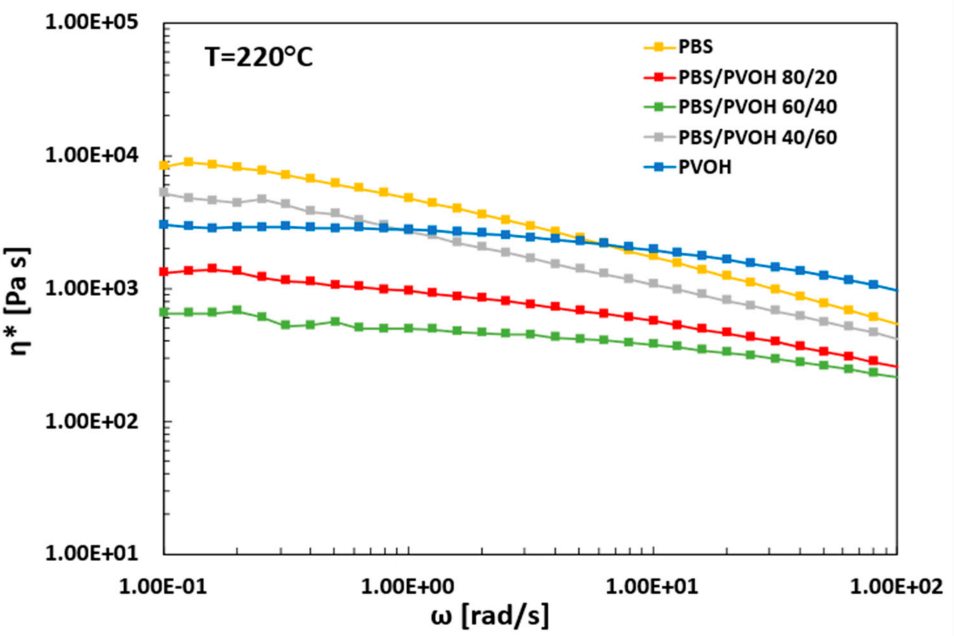

(**a**)

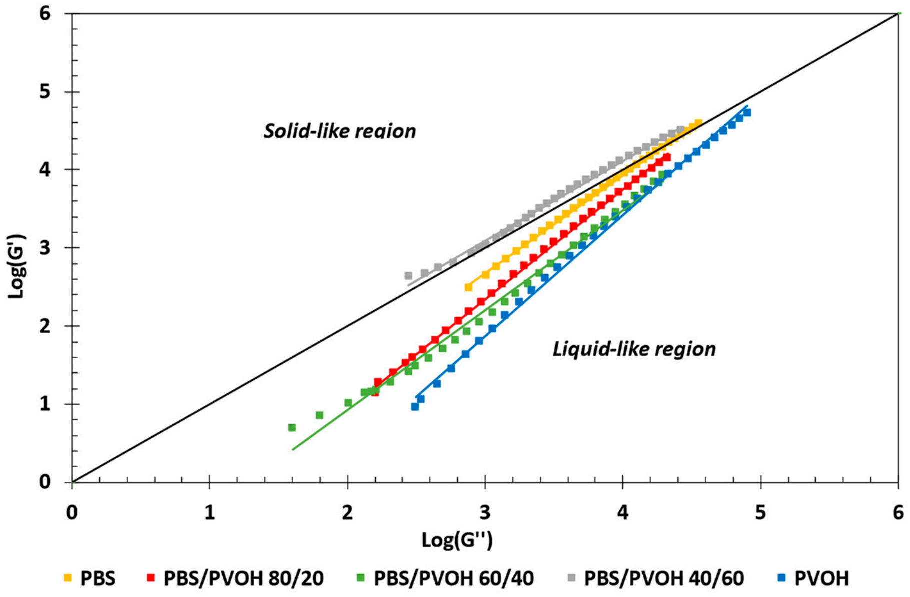

(**b**)

Figure S1. Dynamic rheological response of neat PBS, neat PVOH, and PBS/PVOH blends at 220 °C: (a) complex viscosity as a function of angular frequency and (b) Han plot expressed as log G′ versus log G″

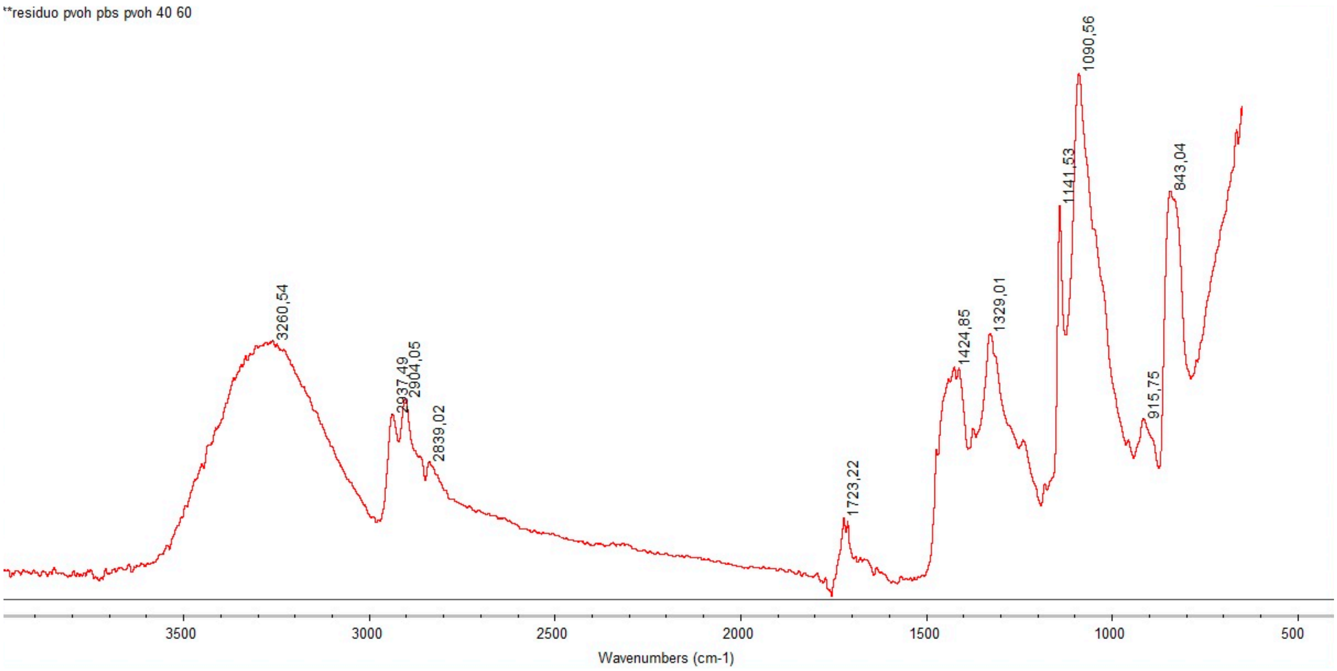

Figure S2. FTIR spectrum of the insoluble residue recovered from the PBS/PVOH 40/60 blend. The characteristic absorption bands confirm that the insoluble fraction consists essentially of PVOH, showing the main signals attributable to –OH, C–H, and C–O groups typical of poly(vinyl alcohol).

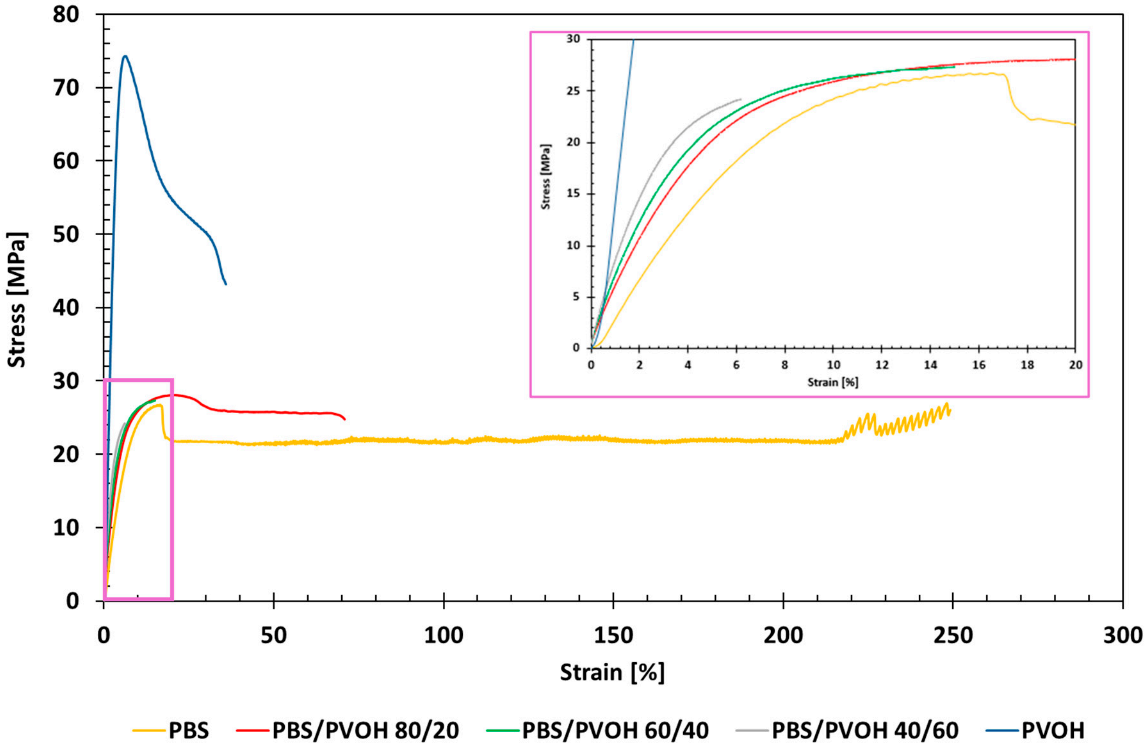

Figure S3. Representative stress–strain curves of neat PBS, neat PVOH, and processable PBS/PVOH blend films measured in the machine direction according to ASTM D882.
